# *stg* fimbrial operon from *S.* Typhi STH2370 contributes to association and cell disruption of epithelial and macrophage-like cells

**DOI:** 10.1186/s40659-015-0024-9

**Published:** 2015-07-07

**Authors:** Liliana Berrocal, Juan A Fuentes, A Nicole Trombert, Matías R Jofré, Nicolás A Villagra, Luis M Valenzuela, Guido C Mora

**Affiliations:** Laboratorio de Microbiología, Facultad de Ciencias Biológicas, Universidad Andres Bello, República 217, Santiago, Chile; Escuela de Medicina, Facultad de Medicina, Universidad Finis Terrae, Santiago, Av. Pedro de Valdivia 1509, Providencia, Santiago, Chile; Centro de Genómica y Bioinformática, Facultad de Ciencias, Universidad Mayor, Santiago, Camino La Pirámide 5750, Huechuraba, Santiago, Chile; Laboratorio de Microbiología, Facultad de Medicina, Universidad Andres Bello, República 313, Santiago, Chile

**Keywords:** *Salmonella* Typhi, *stg*, Fimbriae, Adherence, Association bacteria—eukaryotic cells

## Abstract

**Background:**

*Salmonella enterica* serovar Typhi (*S.* Typhi) *stg* operon, encoding a chaperone/usher fimbria (CU), contributes to an increased adherence to human epithelial cells. However, one report suggests that the presence of the Stg fimbria impairs the monocyte—bacteria association, as deduced by the lower level of invasion to macrophage-like cells observed when the *stg* fimbrial cluster was overexpressed. Nevertheless, since other CU fimbrial structures increase the entry of *S.* Typhi into macrophages, and considering that transcriptomic analyses revealed that *stg* operon is indeed expressed in macrophages, we reassessed the role of the *stg* operon in the interaction between *S.* Typhi strain STH2370 and human cells, including macrophage-like cells and mononuclear cells directly taken from human peripheral blood.

**Results:**

We compared *S.* Typhi STH2370 WT, a Chilean clinical strain, and the *S.* Typhi STH2370 Δ*stg* mutant with respect to association and invasion using epithelial and macrophage-like cells. We observed that deletion of *stg* operon reduced the association and invasion of *S.* Typhi, in both cellular types. The presence of the cloned *stg* operon restored the WT phenotype in all the cases. Moreover, we compared *Salmonella enterica* sv. Typhimurium 14028s (*S.* Typhimurium, a serovar lacking *stg* operon) and *S.* Typhimurium heterologously expressing *S.* Typhi *stg*. We found that the latter presents an increased cell disruption of polarized epithelial cells and an increased association in both epithelial and macrophage-like cells.

**Conclusions:**

*S.* Typhi *stg* operon encodes a functional adhesin that participates in the interaction bacteria—eukaryotic cells, including epithelial cells and macrophages-like cells. The phenotypes associated to *stg* operon include increased association and consequent invasion in bacteria—eukaryotic cells, and cell disruption.

**Electronic supplementary material:**

The online version of this article (doi:10.1186/s40659-015-0024-9) contains supplementary material, which is available to authorized users.

## Background

An essential step in the successful colonization and production of disease by microbial pathogens is their ability to adhere to host cells surfaces [1]. Most pathogens possess many molecules (called adhesins) that mediate host adherence. Often these molecules are synergistic in their function, thereby enhancing adherence, while others appear to be functionally redundant [2]. Fimbriae, also called pili, are important adhesins that facilitate host tissue colonization in a large number of pathogens. According to their biosynthetic pathway, fimbriae can be classified into five major groups: curli, type IV pili, type III secretion needle, type IV secretion pili, and chaperone/usher fimbriae (CU fimbriae) [3–6]. Genome analyses of *Enterobacteriaceae* family members, including the host-specific *Salmonella enterica* serovar Typhi (*S.* Typhi), revealed that there are at least twelve fimbria operons involved in the CU dependent pathway but only few of them have been characterized to date [7]. *Salmonella enterica* (*S. enterica*) serovars, normally acquired by ingesting contaminated water or food, have undergone mutations and horizontal genetic transfer, resulting in unique combinations of fimbrial operons. This presumably contributes to genus evolution and to the rising of different adhesive properties [7, 8]. Actually, sequencing of *S.* Typhi genome reveals twelve operons encoding fimbriae of the CU assembly pathway (i.e. *sef, fim, saf, tcf, bcf, sta, stb, stc, std, ste, stg*, and *sth*), while five of these operons (i.e. *sef, tcf, sta, ste,* and *stg*) are absent from the generalist *S. enterica* serovar Typhimurium (*S.* Typhimurium) [7]. Thus, the differences found between *S.* Typhi and *S.* Typhimurium, including the host-specificity, might be based on the bacteria—host cell interplay. This interaction depends, at least in part, on specific sets of fimbriae contributing to the development of the disease [7].

Among CU fimbrial operons present in *S.* Typhi and absent from *S.* Typhimurium, *stg* has caught our interest. This operon is constituted by four open reading frames known as *stgA* (main fimbrial subunit), *stgB* (chaperone), *stgC* (outer membrane usher) and *stgD* (adhesion tip). Previously, it was reported that *stgC* (STY3920) contains a premature stop codon that disrupts the predicted open reading frame (ORF) encoding the usher; therefore *stgC* was considered a pseudogene [9]. Nevertheless, the *stg* operon seems to encode a functional fimbria since a *S.* Typhi Δ*stg* mutant exhibits an decreased adherence to human epithelial cells compared with the WT [10]. In contrast, in the same report the authors suggest that the presence of the Stg fimbria impairs the macrophage-like—bacteria association, as deduced by the lower level of invasion to monocytes observed when the *stg* fimbrial cluster was overexpressed [10]. Nevertheless, it has been reported that other CU fimbrial structures increase the entry of *S.* Typhi into macrophages/monocytes [11]. Most importantly, transcriptomic analyses revealed that *stg* operon is indeed expressed in macrophages, suggesting that this operon might be participating in the interaction with these cells. These data prompted us to reassess the role of the *stg* operon with respect to the interaction between *S.* Typhi and human cells, including macrophage-like cells and mononuclear cells directly taken from human peripheral blood.

In this work, we determined that the *stg* operon contributed to increase association of bacteria and both epithelial and macrophage-like cells. Moreover, *stg* operon contributes to cell invasion and epithelial cell disruption, strongly suggesting that the Stg fimbria are actively participating in different steps of *S.* Typhi infection process.

## Results

### The *stg* operon contributes to the association, invasion, and to an increased permeability of HEp-2 human epithelial cells in *S. enterica*

**Figure 1:**
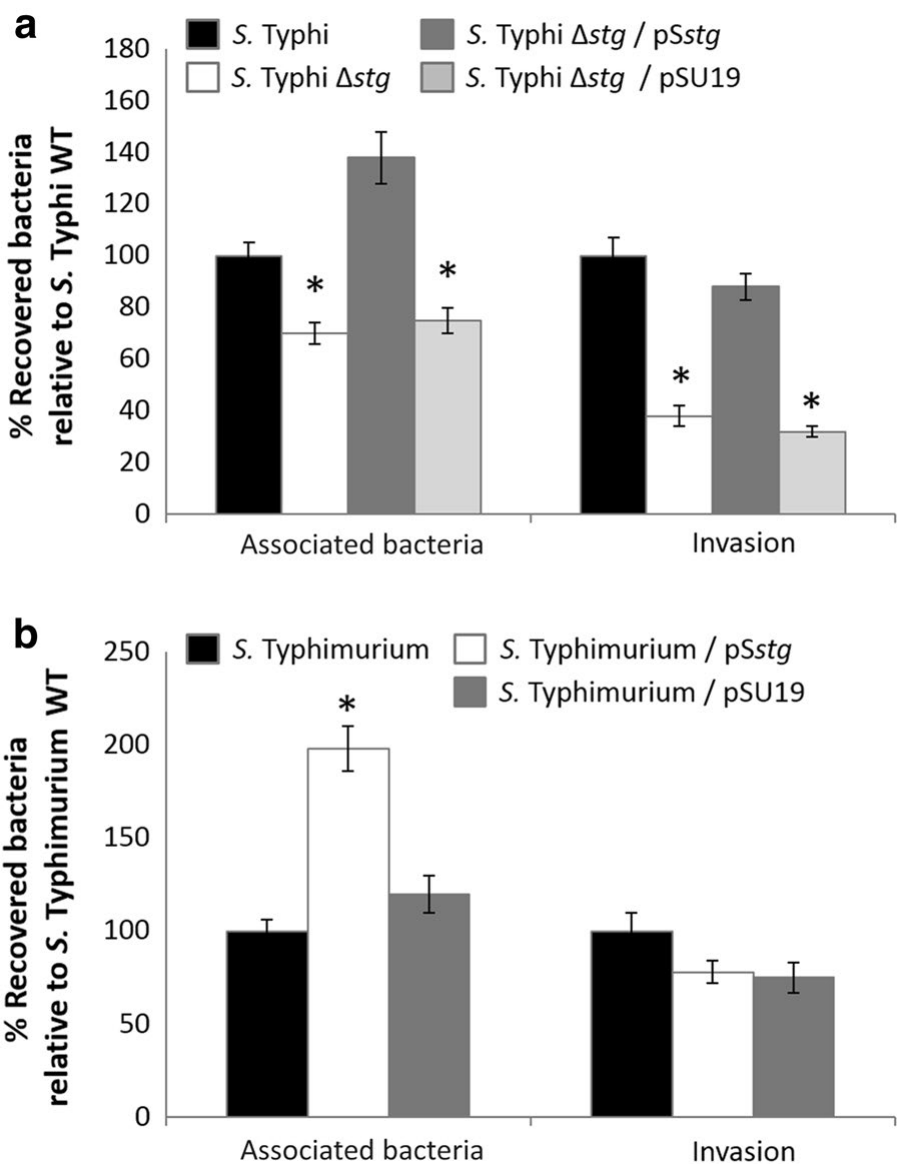
Association and invasion of HEp-2 epithelial cells. The strains used include *S.* Typhi STH2370 WT (*black*), *S.* Typhi STH2370 Δ*stgABCD*::FRT (Δ*stg*) (*white*), *S.* Typhi STH2370 Δ*stgABCD*::FRT/pS*stg* (Δ*stg*/pS*stg*) (*dark grey*), and *S.* Typhi STH2370 Δ*stgABCD*::FRT/pSU19 (Δ*stg*/pSU19) (*light grey*) (**a**); and *S.* Typhimurium 14028s WT (*black*), *S.* Typhimurium 14028s WT/pS*stg* (*white*), and *S.* Typhimurium 14028s/pSU19 (*dark grey*) (**b**). The figure shows values expressed as the mean ± standard deviation of three full biological replicates, each time in technical triplicate. *p < 0.05 (Student’s-test) compared with the WT in the corresponding group.

Considering that one of the first steps in the *S. enterica* infection involves the interaction with human epithelial cells, the contribution of the *stg* operon to cell adherence was assessed using HEp-2 cells. For that, the strains to be tested were cultured in LB to OD_600_ = 0.2 in microaerophilia without shaking prior to determining the number of bacteria associated to eukaryotic cells and the number of bacteria that invaded as described in “Methods”. Associated bacteria can be defined as adherent bacteria plus bacteria that invaded during the early stage of the interaction between bacteria and eukaryotic cells. As observed in Figure 1a, *S.* Typhi Δ*stgABCD* (i.e. Δ*stg*) exhibited a significantly lower level of association to HEp-2 compared to the otherwise isogenic *S.* Typhi STH2370 WT, a highly virulent Chilean strain [12]. The complementation with the *S.* Typhi *stg* whole operon cloned into the pSU19 plasmid (pS*stg*) restored the WT phenotype, whereas the empty vector pSU19 exerted no effect (Figure 1a; Additional file 1: Table S1). On the other hand, *S*. Typhimurium naturally lacks the *stg* operon [7]. Thus, to test the contribution of the *stg* operon in a heterologous system, we transformed pS*stg* into *S.* Typhimurium 14028s WT prior to testing the bacterial association to the HEp-2 cells. As shown in Figure 1b (and Additional file 1: Table S1), *S.* Typhimurium 14028s WT/pS*stg* exhibited an increased association compared with the respective *S.* Typhimurium 14028s WT.

Considering that an increased association between bacteria and epithelial cells conceivably may affect the subsequent bacterial internalization, we assessed whether *stg* was implicated in the invasion of epithelial cells. The invasion is a critical step in the normal infection cycle of *S. enterica*. Since the invasion is promoted by effectors injected into host cells through a type-III secretory system, an increased association bacteria—epithelial cell might affect this process. To test this hypothesis, we performed a gentamicin protection assay comparing the invasion rates of *S.* Typhi STH2370 WT and *S.* Typhi Δ*stg*. As shown in Figure 1a, *S.* Typhi Δ*stg* presented an impaired invasion compared with the WT strain, consistent with the results obtained for the association bacteria—epithelial cells. Again, the pS*stg* plasmid, and not the vector alone, restored the WT phenotype (Figure 1a, Additional file 1: Table S1). Nevertheless, when invasion efficiency was calculated by determining the ratio of invaded/associated bacteria using the raw data (Additional file 1: Table S1), no significant differences were observed. This result suggests that the decreased invasion is a consequence of a decreased association, probably mediated by an effect in adherence, and not to an effect in invasion specifically. On the other hand, the pS*stg* plasmid apparently produced no effects in the invasion of the *S.* Typhimurium heterologous strain (Figure 1b), showing that the *stg* operon is not specifically contributing to the invasion of *S.* Typhimurium 14028s under the tested conditions.

**Figure 2:**
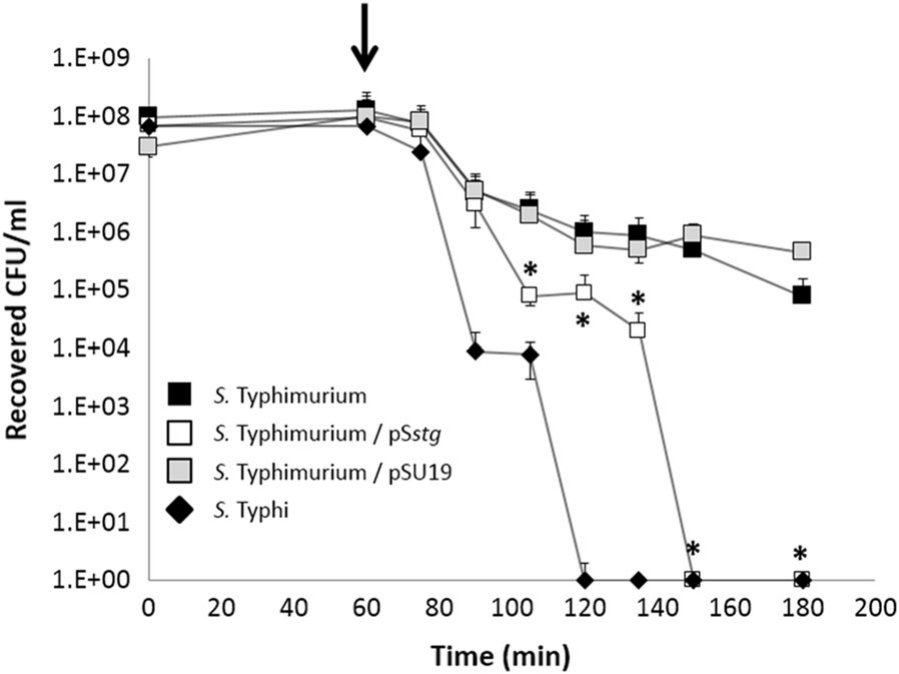
Cell permeability assay of *S.* Typhi STH2370 WT, and *S.* Typhimurium 14028s WT and derivatives, through H-T29 human cell line monolayers. The *arrow* indicates the time at which gentamicin was added. The experiments were performed in three full biological replicates, each time in technical triplicate. The values are expressed as the mean ± standard deviation of three independent experiments. *p < 0.005 (Student’s-test).

*S.* Typhi exhibits a high cytotoxicity towards eukaryotic cells compared to *S.* Typhimurium, clearly affecting the permeability of the infected cells. This phenotype can be explained because of the presence of cytolytic factors in the serovar Typhi and/or because of the presence of proteins that diminishes the cell cytotoxicity in the serovar Typhimurium [13–15]. Since the *stg* operon is only present in the serovar Typhi and absent from the serovar Typhimurium, we assessed whether the presence of the *stg* operon can also affect the cell permeability of a monolayer of epithelial cells. For that, polarized HT-29 monolayers were cultured in Transwells and incubated for 1 week to allow cell polarization with an apical zone (upper chamber) and a basolateral zone (lower chamber). Cell polarization was confirmed by a gradual increase of transepithelial resistance (TER) (data not shown). The polarized monolayers were subsequently infected with *S.* Typhimurium 14028s WT, *S.* Typhimurium 14028s WT/pS*stg*, and *S.* Typhimurium 14028s WT/pSU19. As control, we used *S*. Typhi STH2370 WT. The infected monolayers were used to perform a modified transepithelial migration assay that included addition of gentamicin (after 1 h of infection) into the upper chamber as previously described [14, 15]. As shown in Figure 2, the recovered CFU/mL represented bacteria that migrated to the lower chamber and survived the presence of the gentamicin leaking through the cell monolayer. If bacteria disrupt the integrity of the monolayer, gentamicin will leak through from the upper chamber to the lower chamber, killing bacteria in the lower chamber and decreasing the recovered CFU/mL. On the other hand, if the monolayer is not disrupted, the recovered CFU/mL should remain essentially constant over the same time course since gentamicin cannot permeate through cellular membranes [16]. As observed in Figure 2, the recovered CFU/mL corresponding to *S.* Typhimurium 14028s presented a slight decline over the time course of the assay (black squares), showing that the monolayer integrity is not largely affected by bacteria in this case, accordingly to previous published studies [14, 15]. In contrast, the CFU/mL of *S.* Typhi STH2370 recovered from the lower chamber abruptly decreased after the gentamicin addition until they became undetectable, showing that the gentamicin leaked into the lower chamber due to a monolayer disruption (black diamonds). Most importantly, when *S.* Typhimurium 14028s was complemented with the *S.* Typhi *stg* operon (pS*stg*, white squares) we observed that the corresponding recovered CFU/mL clearly decreased, marking a sharp difference with the otherwise isogenic *S.* Typhimurium 14028s WT strain and resembling the *S.* Typhi phenotype (compare the black squares and white squares) (Figure 2). All these results were corroborated by measuring the transepithelial electrical resistance (TER) of the infected HT-29 monolayer as previously described [14] (data not shown).

All these results together show that the *stg* operon contributes to increase the interaction bacteria—epithelial cell and the invasion in *S.* Typhi. Moreover, when heterologously expressed, *stg* contributes to increase the adherence, and cell disruption in *S.* Typhimurium.

### The *stg* operon contributes to increase the association of bacteria and human macrophage-like cells in *S. enterica*

**Figure 3:**
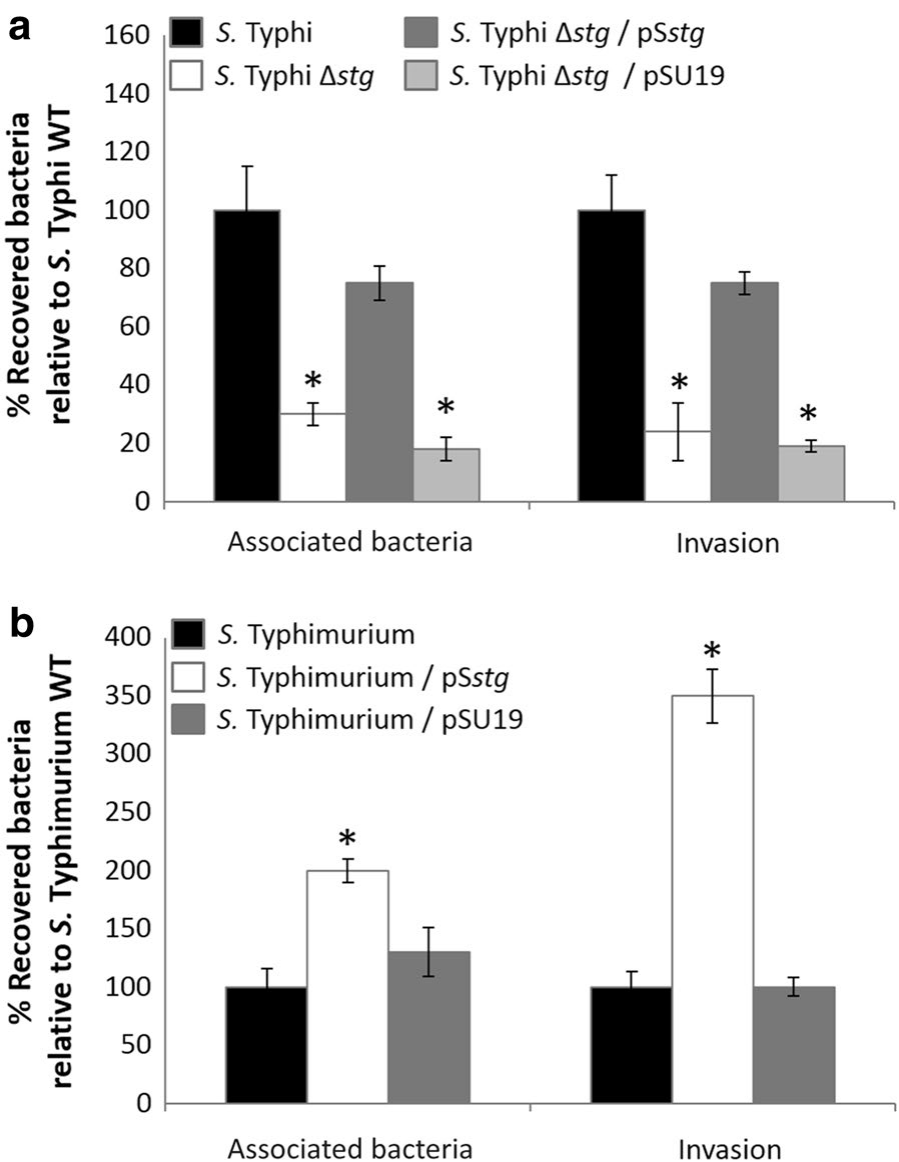
Association and invasion of monocytes U937 (**a**) or mononuclear cells directly extracted from human blood (**b**). The strains used include *S.* Typhi STH2370 WT (*black*), *S.* Typhi STH2370 Δ*stgABCD*::FRT (Δ*stg*) (*white*), *S.* Typhi STH2370 Δ*stgABCD*::FRT/pS*stg* (Δ*stg*/pS*stg*) (*fark grey*), and *S.* Typhi STH2370 Δ*stgABCD*::FRT/pSU19 (Δ*stg*/pSU19) (*light grey*) (**a**); and *S.* Typhimurium 14028s WT (*black*), *S.* Typhimurium 14028s WT/pS*stg* (*white*), and *S.* Typhimurium 14028s/pSU19 (*dark grey*) (**b**). The figure shows values expressed as the mean ± standard deviation of three full biological replicates, each time in technical triplicate. *p < 0.05 (Student’s-test) compared with the WT in the corresponding group.

Macrophage-like cells play an important role in the dissemination of *Salmonella enterica* in the systemic phase of the disease [9]. In a previous report, it has been stated that deletion of *stg* increased uptake of serovar Typhi by human macrophages, and overexpression of *stg* operon in *S.* Typhi and *S.* Typhimurium strains reduced phagocytosis by human macrophages [10]. In that study, *S.* Typhi strain ISP1820 and derivatives were grown to stationary phase under aerobic conditions, prior to performing the infection [10]. Nevertheless, other studies reported that the *stg* operon increases its expression during an infection of macrophages [17, 18], suggesting that this operon may contribute to *S.* Typhi invasion in these cells. Considering that other CU fimbriae have an active role in bacterial invasion of host phagocytic cells [11, 19], we reassessed the role of the *stg* operon in the macrophage-like cell interaction. The first approach was to determine the role of *stg* in the bacterial association to human macrophage-like cells (monocytes). For that, the human monocyte cell line U937 was infected with *S.* Typhi STH2370 WT, *S.* Typhi STH2370 Δ*stg*, *S.* Typhi STH2370 Δ*stg*/pS*stg*, or *S.* Typhi STH2370 Δ*stg*/pSU19, previously cultured in LB to OD_600_ = 0.2 under microaerophilic conditions, to perform the adherence and invasion assays. As shown in Figure 3a and in Additional file 1: Table S2, *S.* Typhi Δ*stg* exhibited a significant impaired association to U937 cells compared to the WT strain. Trans-complementing with the pS*stg* plasmid reverted the mutant phenotype (Figure 3a). Considering that a lower association could lead to a decreased bacterial invasion, we determined the bacterial uptake by U937 monocytes using the gentamicin protection assay. As observed in Figure 3a, the *S.* Typhi Δ*stg* also presented a decreased invasion compared with the WT. Again, when invasion efficiency was calculated (see data in Additional file 1: Table S2), no significant differences were observed, suggesting that the contribution of *stg* to the invasion depends on the association (plausibly on the adherence) in this case.

To assess the contribution of the *stg* operon in macrophage-like—bacteria interaction in an independent way, we performed association and invasion assays using *S.* Typhimurium heterologously expressing *S.* Typhi *stg* from the pS*stg* plasmid. In this case, we tested the *S.* Typhimurium/pS*stg* with a mononuclear cell fraction obtained directly from human peripheral blood isolated with HISTOPAQUE^®^-1077. As observed in Figure 3b and in Additional file 1: Table S3, the presence of the *stg* operon in *S.* Typhimurium significantly contributes to the bacterial uptake compared with the control. In all cases, the empty vector exerted no effects (Figure 3). The invasion efficiency (Additional file 1: Table S3) presented no significant differences among the strains, suggesting that also in this case the contribution of *stg* to invasion is dependent on the bacteria—eukaryotic cells association.

All these results together show that the *stg* operon contributes to the macrophage-like—bacteria interaction, contributing to the association and the invasion in a line of human monocytes (i.e. U937) and in mononuclear cells directly extracted from human blood.

## Discussion

The adherence is a critical process for host colonization and disease production in several pathogens, including *S. enterica* [1]. In this work, we found that *stg* operon contributes to adherence/invasion of *S.* Typhi; and to adherence/invasion, and cell disruption when heterologously expressed in *S.* Typhimurium. These findings strongly suggest that *stgABCD* encode a functional fimbria that is able to assemble under the growth conditions used. Nevertheless, the *stgC* gene, encoding the putative usher for the fimbria assembly, contains an internal stop codon reported for *S.* Typhi CT18; therefore it has been considered as a pseudogene (i.e. non-functional) [7]. We sequenced an internal fragment of the *stgC* gene of the Chilean clinical strain *S.* Typhi STH2370 [12], a highly virulent isolate, and we observed that the stop codon is at the same position observed in the sequenced strain *S.* Typhi CT18 (data not shown). A non-functional *stgC* could preclude the fimbrial assembly, affecting the phenotypes observed in this work. However, despite the presence of the single internal stop codon in *stgC*, a fully functional StgC protein might be produced. In some cases, single premature stop codons can be hopped by ribosomes, allowing a normal protein synthesis in *S. enterica* [20], similarly to some phage genes containing TGA premature stop codons [21]. This same mechanism might occur in *stgC* gene to produce a functional usher protein. Another explanation could be the complementation of the StgC function (i.e. usher) by functional ushers from other chaperon-usher operons found in *S. enterica*, due to the high identity found among them [7]. Nevertheless, more experiments have to be performed in order to test these hypotheses.

Here, we also showed that the *stg* operon, present in *S.* Typhi and absent from *S.* Typhimurium, contributes to the interaction between *S. enterica* and host cells, including epithelial cells and macrophage-like cells. In the epithelial cell line HEp-2, we observed that *stg* contributed to the bacterial association to epithelial cells, suggesting that *stg* could be important in the first steps of *S. enterica* infection, accordingly to previously reported studies that showed an impaired adherence of *S.* Typhi Δ*stg* mutant to the INT-407 epithelial cell line [10]. We also found that the increased association due to the presence of the *stg* operon is accompanied with an improved invasion of HEp-2 cells. Nevertheless, Forest et al. showed that apparently the *stg* operon is not contributing to this process [10], similarly to the observed with *S.* Typhimurium heterologously expressing *stg* (Figure 1b). This difference could be explained by the fact that we cultured bacteria under microaerophilic conditions prior to performing the invasion assay, as previously described [16]. In contrast, Forest et al. [10] cultured bacteria to stationary phase with aeration. The microaerophilic condition is an important inducer of the invasion process in *S. enterica* (unpublished results and [22]). Thus, the bacterial culture condition seems to be a key factor to assess the interaction between bacteria and eukaryotic cells. On the other hand, the null contribution of *stg* to the *S.* Typhimurium invasion could explain why this serovar lacks the *stgABCD* operon [7].

Cell permeability in HT-29 epithelial cells was also affected by the presence of the *stg* operon in *S*. Typhimurium (Figure 2), plausibly due to cell disruption [14]. Some authors have suggested that *S.* Typhi has been selected for an increased disruptive ability towards epithelial cells compared to *S.* Typhimurium in order to perform a successful systemic/deeper infection in humans [14, 15]. Nevertheless, we found no differences between *S.* Typhi WT and *S.* Typhi Δ*stg* with respect to the cells disruption (data not shown). This result may be explained by the fact that *S.* Typhi presents several mechanisms to disrupt cells. Actually, *S.* Typhi presents other factors contributing to increase the ability to disrupt epithelial cells. Some of these factors, as well as *stg*, are absent from *S.* Typhimurium, including *hlyE*, and *sseJ* and *sopD2* pseudogenes [13–15]. However, how *stg* is improving cell disruption in *S.* Typhimurium is unknown. We speculate that *stg* contributes to this phenotype by increasing the bacteria—cell contact. Thus, the results showed in this paper indicate that the *stg* fimbrial operon has a role in association, invasion, and in disruption of epithelial cells in *S. enterica*.

During the *S.* Typhi infectious process, bacteria interact with phagocytic cells that play a crucial role in bacterial systemic dissemination and possibly in host specificity [10, 23]. Our results with monocyte U937 cells and with human mononuclear cells suggested a role of the *stg* operon in bacterial association and invasion of phagocytic cells. These results are consistent with works showing the expression of *stg* fimbrial genes in human macrophages [17, 18]. Other chaperon-usher fimbriae, such as *sef* operon, proved to be involved in bacterial interaction with phagocytic cells during peritoneal infection in *Salmonella enterica*, reinforcing the fact that this kind of fimbriae could contribute to the systemic spreading [11]. In contrast, Forest et al. [10] reported that loss of *stg* results in increased phagocytosis of serovar Typhi by THP-1 human macrophages-like cells. Again, this apparent contradiction to our findings could be explained by the bacterial growth conditions used before infecting monocytes. Forest et al. used bacteria grown overnight at 37°C before infection of cells [10], while we used bacteria grown to OD_600_ = 0.2 under microaerophilic conditions without shaking, a condition promoting an efficient invasion to macrophages [24, 25]. Finally, it is important to remark that Forest et al. [10] worked with *S.* Typhi strain ISP1820, whereas, in this manuscript, we report a study performed with *S.* Typhi strain STH2370 [12], a Chilean clinical isolate. Considering that *S.* Typhi STH2370 presents some genomic differences compared with other standard *S.* Typhi strain such as CT18 and Ty2 (unpublished data and [12, 26, 27]), it is also possible that the differences between our results and previous data are due to the bacterial strains tested.

As stated above, *stg* is contributing to bacterial interaction with both epithelial cells and macrophages-like cells, strongly suggesting that the *stg* operon could participate in the intestinal step of infection as well as in the systemic spread. It has been described other virulence factors not circumscribed to a unique stage of the infection. For example, *Salmonella* SipA, encoded by SPI-1 enhances entry to intestinal epithelial cells by promoting actin polymerization, but also contributing to proliferation of *S. enterica* within macrophages [28]. Moreover, other genes belonging to SPI-1 such as *prg*, *sip*, *spa*, and *inv* genes increase their expression inside the gut and also during early stages of bacterial uptake by human macrophages [18]. The possible role of *stg* operon in both stages of *S.* Typhi infection (i.e. intestinal and systemic) suggests that it may encode an important factor for the pathogenesis of this human-specific pathogen.

## Conclusions

*S.* Typhi *stg* operon, encoding a functional adhesin, contributes to the interaction of bacteria and eukaryotic cells, including epithelial and macrophages-like cells. The phenotypes associated to *stg* operon include stg operon include increased association and consequent invasion in bacteria—eukaryotic cells, and cell disruption.

## Methods

### Culture conditions

Clinical isolate of *S.* Typhi strain STH2370, a highly virulent strain from the Infectious Diseases Hospital Lucio Córdova in Santiago, Chile. The *S.* Typhimurium strains used in this study are derivatives of the parental wild-type strain ATCC14028s. The strains were routinely grown in Luria–Bertani (LB) broth (Bacto tryptone, 10 g/L; Bacto yeast extract, 5 g/L; and NaCl, 5 g/L) at 37°C with shaking. For assessing bacteria—eukaryotic cells association and for the invasion assays, bacteria were cultured in LB broth at 37°C in microaerophilia as previously described [16]. Culture media were supplemented with chloramphenicol (20 µg/mL), ampicillin (100 µg/mL) or kanamycin (50 µg/mL) when required. Solid media contain Bacto agar (Merck) at 15 g/L.

### Construction of mutants

*S.* Typhi Δ*stgABCD*::FRT (or simply named *S.* Typhi Δ*stg*) was constructed using the Red-Swap procedure as described [29]. The primers used in the lambda red recombination system correspond to WNstgA (AATGAAACATCGGGATATATAATATATCAATAGAGTTATACATATGAATATCCTCCTTAG) and WCstgD (TTGATATGACTTATTTTGTAATTAGATTAGTTCGTTATTTTGTAGGCTGGAGCTGCTTCG).

### PCR amplifications and plasmid construction

PCR amplifications were performed using an Eppendorf thermal cycler and *Accuprime Taq* DNA polymerase (Invitrogen). Reaction mixtures contained 1X PCR buffer, 1.5 mM MgCl_2_, each dNTP (200 μM), primers (1 μM), 100 ng of template DNA, and 2 U polymerase. Standard conditions for amplification were 30 cycles at 96°C for 40 s, 55°C for 45 s, and 72°C for 2 min, followed by a final extension step at 72°C for 10 min. Template *S.* Typhi STH2370 (WT) chromosomal DNA was prepared as described [30]. The pS*stg* plasmid was constructed by digesting the pG*stg* with *Eco*RI and cloning the fragment that contained the *stg* operon into the *Eco*RI site in mid-copy vector pSU19. Primers used for *stg* operon amplification were NstgA (AGCGACAACATGCACATCAT), and CstgD (CAGGGCCGTAGTTCTTGAT). Products generated by PCR amplification were resolved in 0.8% agarose gels.

### Determination of bacteria: eukaryotic cell association and invasion assays

Macrophage-like human cell line (U937), and the epithelial cell lines (HEp-2 and HT-29) were maintained in RPMI 1640 (Invitrogen) containing 10% fetal calf serum pre-treated for 30 min at 60°C, at 37°C in a 5% CO_2_/95% air mixture. Peripheral mononuclear cells were obtained from healthy people using HISTOPAQUE^®^-1077 according the protocol described by Boyum et al. [31]. For assessing the bacteria—epithelial cell association, HEp-2 cells were seeded at a concentration of 5 × 10^5^ cells per well in 96-well microtiter plates. Bacteria were grown in microaerophilia as previously described [16] in static conditions to an OD_600_ = 0.2 prior to being added to a cell monolayer at a MOI of 100:1. After incubation for 1 h at 37°C, the infected cells were washed five times with sterile PBS. The eukaryotic cells were disrupted with sodium deoxycholate 0.5% prior to determining the number of bacteria associated to the eukaryotic cells by bacterial plate counting (CFU). The level of associated bacteria was expressed as a percentage of the initial inoculum. For monocytes cell association assays, U937 cells were seeded at a concentration of 5 × 10^5^ cells in 1.5 mL tubes. Bacteria were grown in microaerophilia as previously described [16] in static conditions to an OD_600_ = 0.2 prior to being added to macrophages at a MOI of 100:1. The mixture U937 and bacteria was centrifuged for 5 min at 1,000×*g* to synchronize the bacteria—cell interaction. After incubation for 1 h at 37°C, the infected cells were washed five times with sterile PBS prior to adding 0.5% deoxycholic acid sodium salt in PBS. Bacteria associated to eukaryotic cells were determined by bacterial plate counting (CFU). The level of association was expressed as a percentage of the initial inoculum. To determine the invasion rate to HEp-2 epithelial cells, the U937 macrophage-like cells, or mononuclear cells, the gentamicin protection assay was performed as previously described [13].

### Transepithelial migration assay

Colon carcinoma HT29 cells were grown to confluence on 3.0 mm pore-size filters (“Transwells”, Millicell^®^, Millipore) in glucose-free RPMI medium (Gibco) for 2 weeks at 37°C and 5% of CO_2_ to allow cell polarization, which was subsequently confirmed by a gradual increase of transepithelial resistance (TER). Transwells with cellular monolayers were inoculated apically with 400 μL of bacterial cultures at approximately 1 × 10^7^ CFU/mL previously grown in microaerophilia [16] to and OD_600_ = 0.2 in LB. After 1 h incubation at 37°C to allow bacterial uptake by the human cells, monolayers were washed once with sterile PBS and 400 µL of glucose-free RPMI medium containing gentamicin (50 µg/mL) was added to eliminate extracellular bacteria. Aliquots (20 μL) from the basal compartment (lower chamber) were then collected every 15 min and up to 3 h of incubation to monitor the number of bacteria that crossed the cell monolayer as determined by CFUs count on LB agar plates. All the results obtained with the transepithelial migration assay were corroborated by measuring the transepithelial electrical resistance as previously described [14].

### Consent

All the human subjects that donated blood presented an informed consent to participate in this study.

### Statistics

All results are expressed as the mean ± SD of an individual experiment performed in triplicate. *p* values were calculated according to Student’s t test, and a value of *p* < 0.05 was considered to be statistically significant.

## Additional file

Additional file 1: Association or invasion of different eukaryotic cells, including HEp-2 (epithelial cells), U937 (macrophage-like cells), and mononuclear cells directly extracted from human blood. The bacterial strains used include *S.* Typhi STH2370 WT, *S.* Typhi STH2370 Δ*stgABCD*::FRT (Δ*stg*), *S.* Typhi STH2370 Δ*stgABCD*::FRT/pS*stg* (Δ*stg*/pS*stg*), and *S.* Typhi STH2370 Δ*stgABCD*::FRT/pSU19 (Δ*stg*/pSU19); and *S.* Typhimurium 14028s WT, *S.* Typhimurium 14028s WT/pS*stg*, and *S.* Typhimurium 14028s/pSU19. The tables show values expressed as the means ± standard deviation of 3 full biological replicates, each time in technical triplicate. *p < 0.05 (Student’s-Test) compared with the WT in the corresponding group.

## Abbreviations

*S. enterica*: *Salmonella enterica*
*S.* Typhi: *Salmonella enterica* serovar Typhi
*S.* Typhimurium: *Salmonella enterica* serovar Typhimurium
*stg*: *stgABCD* operon

## References

[1] Godaly G, Frendeus B, Proudfoot A, Svensson M, Klemm P, Svanborg C (1998). Role of fimbriae-mediated adherence for neutrophil migration across *Escherichia coli*-infected epithelial cell layers. Mol Microbiol..

[2] Wagner C, Hensel M (2011). Adhesive mechanisms of *Salmonella enterica*. Adv Exp Med Biol..

[3] Thanassi DG, Saulino ET, Hultgren SJ (1998). The chaperone/usher pathway: a major terminal branch of the general secretory pathway. Curr Opin Microbiol..

[4] Wu H, Fives-Taylor PM (2001). Molecular strategies for fimbrial expression and assembly. Crit Rev Oral Biol Med Off Publ Am Assoc Oral Biol..

[5] van der Woude MW, Baumler AJ (2004). Phase and antigenic variation in bacteria. Clin Microbiol Rev..

[6] Waksman G, Hultgren SJ (2009). Structural biology of the chaperone-usher pathway of pilus biogenesis. Nat Rev Microbiol..

[7] Townsend SM, Kramer NE, Edwards R, Baker S, Hamlin N, Simmonds M (2001). *Salmonella enterica* serovar Typhi possesses a unique repertoire of fimbrial gene sequences. Infect Immun..

[8] Baumler AJ (1997). The record of horizontal gene transfer in *Salmonella*. Trends Microbiol..

[9] Edwards RA, Matlock BC, Heffernan BJ, Maloy SR (2001). Genomic analysis and growth-phase-dependent regulation of the SEF14 fimbriae of *Salmonella enterica* serovar Enteritidis. Microbiology..

[10] Forest C, Faucher SP, Poirier K, Houle S, Dozois CM, Daigle F (2007). Contribution of the *stg* fimbrial operon of *Salmonella* enterica serovar Typhi during interaction with human cells. Infect Immun..

[11] Edwards RA, Schifferli DM, Maloy SR (2000). A role for *Salmonella* fimbriae in intraperitoneal infections. Proc Natl Acad Sci USA..

[12] Valenzuela C, Ugalde JA, Mora GC, Álvarez S, Contreras I, Santiviago CA. Draft genome sequence of *Salmonella enterica* Serovar Typhi Strain STH2370. Genome Announc. 2014;2(1). doi:10.1128/genomeA.00104-14. 10.1128/genomeA.00104-14 PMC3931366 24558245

[13] Fuentes JA, Villagra N, Castillo-Ruiz M, Mora GC (2008). The *Salmonella* Typhi *hlyE* gene plays a role in invasion of cultured epithelial cells and its functional transfer to *S.* Typhimurium promotes deep organ infection in mice. Res Microbiol..

[14] Trombert AN, Berrocal L, Fuentes JA, Mora GC (2010). *S*. Typhimurium *sseJ* gene decreases the *S.* Typhi cytotoxicity toward cultured epithelial cells. BMC Microbiol..

[15] Trombert AN, Rodas PI, Mora GC (2011). Reduced invasion to human epithelial cell lines of *Salmonella enterica* serovar Typhi carrying *S.* Typhimurium *sopD2*. FEMS Microbiol Lett..

[16] Contreras I, Toro CS, Troncoso G, Mora GC (1997). *Salmonella typhi* mutants defective in anaerobic respiration are impaired in their ability to replicate within epithelial cells. Microbiology..

[17] Faucher SP, Curtiss R, Daigle F (2005). Selective capture of *Salmonella enterica* serovar typhi genes expressed in macrophages that are absent from the *Salmonella enterica* serovar Typhimurium genome. Infect Immun..

[18] Faucher SP, Porwollik S, Dozois CM, McClelland M, Daigle F (2006). Transcriptome of *Salmonella enterica* serovar Typhi within macrophages revealed through the selective capture of transcribed sequences. Proc Natl Acad Sci USA..

[19] Pan Q, Zhang XL, Wu HY, He PW, Wang F, Zhang MS (2005). Aptamers that preferentially bind type IVB pili and inhibit human monocytic-cell invasion by *Salmonella enterica* serovar Typhi. Antimicrob Agents Chemother..

[20] Rodas PI, Trombert AN, Mora GC (2011). A holin remnant protein encoded by STY1365 is involved in envelope stability of *Salmonella enterica* serovar Typhi. FEMS Microbiol Lett..

[21] Goldman E, Korus M, Mandecki W (2000). Efficiencies of translation in three reading frames of unusual non-ORF sequences isolated from phage display. FASEB J Off Publ Fed Am Soc Exp Biol..

[22] Khullar M, Singh RD, Smriti M, Ganguly NK (2003). Anaerobiosis-induced virulence of *Salmonella enterica* subsp. *enterica* serovar Typhimurium: role of phospholipase Cgamma signalling cascade. J Med Microbiol..

[23] Jones BD, Ghori N, Falkow S (1994). *Salmonella Typhimurium* initiates murine infection by penetrating and destroying the specialized epithelial M cells of the Peyer’s patches. J Exp Med..

[24] Singh RD, Khullar M, Ganguly NK (2000). Role of anaerobiosis in virulence of *Salmonella Typhimurium*. Mol Cell Biochem..

[25] Fink RC, Evans MR, Porwollik S, Vazquez-Torres A, Jones-Carson J, Troxell B (2007). FNR is a global regulator of virulence and anaerobic metabolism in *Salmonella enterica* serovar Typhimurium (ATCC 14028s). J Bacteriol..

[26] Parkhill J, Dougan G, James KD, Thomson NR, Pickard D, Wain J (2001). Complete genome sequence of a multiple drug resistant *Salmonella enterica* serovar Typhi CT18. Nature..

[27] Deng W, Liou SR, Plunkett G, Mayhew GF, Rose DJ, Burland V (2003). Comparative genomics of *Salmonella enterica* serovar Typhi strains Ty2 and CT18. J Bacteriol..

[28] Brawn LC, Hayward RD, Koronakis V (2007). *Salmonella* SPI1 effector SipA persists after entry and cooperates with a SPI2 effector to regulate phagosome maturation and intracellular replication. Cell Host Microbe..

[29] Datsenko KA, Wanner BL (2000). One-step inactivation of chromosomal genes in *Escherichia coli* K-12 using PCR products. Proc Natl Acad Sci USA..

[30] Santiviago CA, Toro CS, Hidalgo AA, Youderian P, Mora GC (2003). Global regulation of the *Salmonella enterica* serovar Typhimurium Major Porin, OmpD. J Bacteriol..

[31] Boyum A (1968). Isolation of leucocytes from human blood. A two-phase system for removal of red cells with methylcellulose as erythrocyte-aggregating agent. Scand J Clin Lab Investig Suppl..

