# Supplementary material for: stg fimbrial operon from S. Typhi STH2370 contributes to association and cell disruption of epithelial and macrophage-like cells
Source: Biol Res. 2015 Jul 7;48(1):34. doi: 10.1186/s40659-015-0024-9 (PMC4494162; doi:10.1186/s40659-015-0024-9)
Supplement: Additional file 1: — Association or invasion of different eukaryotic cells, including HEp-2 (epithelial cells), U937 (macrophage-like cells), and mononuclear cells directly extracted from human blood. The bacterial strains used include S. Typhi STH2370 WT, S. Typhi STH2370 ΔstgABCD::FRT (Δstg), S. Typhi STH2370 ΔstgABCD::FRT/pSstg (Δstg/pSstg), and S. Typhi STH2370 ΔstgABCD::FRT/pSU19 (Δstg/pSU19); and S. Typhimurium 14028s WT, S. Typhimurium 14028s WT/pSstg, and S. Typhimurium 14028s/pSU19. The tables show values expressed as the means ± standard deviation of 3 full biological replicates, each time in technical triplicate. *p < 0.05 (Student’s-Test) compared with the WT in the corresponding group. [file 40659_2015_24_MOESM1_ESM.docx]

**Supplementary material**

**Tables**

**Table S1. Association and invasion assays using HEp-2 epithelial cells.**

| **Strain** | **Associated bacteria*** |
| --- | --- |
| *S.* Typhi STH2370 WT | 6.01 ± 0.66 |
| *S.* Typhi Δ*stg* | 4.21 ± 0.54 |
| *S.* Typhi Δ*stg* / pS*stg* | 8.29 ± 0.90 |
| *S.* Typhi Δ*stg* / pSU19 | 4.51 ± 0.60 |
| *S.* Typhimurium 14028s WT | 5.76 ± 0.63 |
| *S.* Typhimurium / pS*stg* | 10.94 ± 1.27 |
| *S.* Typhimurium / pSU19 | 6.34 ± 0.86 |
|  |  |
|  | **Invasion*** |
| *S.* Typhi STH2370 WT | 1.65 ± 0.21 |
| *S.* Typhi Δ*stg* | 0.66 ± 0.13 |
| *S.* Typhi Δ*stg* / pS*stg* | 1.53 ± 0.18 |
| *S.* Typhi Δ*stg* / pSU19 | 0.59 ± 0.08 |
| *S.* Typhimurium 14028s WT | 1.35 ± 0.16 |
| *S.* Typhimurium / pS*stg* | 1.08 ± 0.12 |
| *S.* Typhimurium / pSU19 | 1.01 ± 0.12 |

*** Relative to the input inoculum. These data are represented in Fig. 1 with percentages relative to the corresponding WT.**

**Table S2. Association and invasion assays using U937 macrophage-like cells**

| **Strain** | **Associated bacteria*** |
| --- | --- |
| *S.* Typhi STH2370 WT | 80.03 ± 16.01 |
| *S.* Typhi Δ*stg* | 24.01 ± 6.40 |
| *S.* Typhi Δ*stg* / pS*stg* | 56.02 ± 8.00 |
| *S.* Typhi Δ*stg* / pSU19 | 14.41 ± 7.20 |
|  |  |
|  | **Invasion*** |
| *S.* Typhi STH2370 WT | 0.70 ± 0.11 |
| *S.* Typhi Δ*stg* | 0.18 ± 0.11 |
| *S.* Typhi Δ*stg* / pS*stg* | 0.50 ± 0.05 |
| *S.* Typhi Δ*stg* / pSU19 | 0.13 ± 0.03 |

*** Relative to the input inoculum. These data are represented in Fig. 3A with percentages relative to the corresponding WT.**

**Table S3. Association and invasion assays using macrophage-like cells**

| **Strain** | **Associated bacteria*** |
| --- | --- |
| *S.* Typhimurium 14028s WT | 45.13 ± 4.51 |
| *S.* Typhimurium / pS*stg* | 90.26 ± 4.06 |
| *S.* Typhimurium / pSU19 | 56.41 ± 11.28 |
|  |  |
|  | **Invasion*** |
| *S.* Typhimurium 14028s WT | 0.90 ± 0.08 |
| *S.* Typhimurium / pS*stg* | 3.06 ± 0.23 |
| *S.* Typhimurium / pSU19 | 0.91 ± 0.05 |

*** Relative to the input inoculum. These data are represented in Fig. 3B with percentages relative to the corresponding WT.**
